# An unusual case of cutaneous feline pythiosis

**DOI:** 10.1016/j.mmcr.2019.10.004

**Published:** 2019-10-31

**Authors:** Megan Dowst, Alana Pavuk, Raquel Vilela, Camila Vilela, Leonel Mendoza

**Affiliations:** a South Orlando Animal Hospital, Orlando, FL, 32837, USA; b Antech Diagnostics, Durham, NC, 27713, USA; c Biomedical Laboratory Diagnostics, Michigan State University, East Lansing, MI, 48824, USA; d Microbiology and Molecular Genetics, Michigan State University, East Lansing, MI, 48824, USA

**Keywords:** Feline, Cat, Pythiosis, Pythium insidiosum, Oomycota

## Abstract

Pythiosis is frequently reported in dogs and horses inhabiting tropical, subtropical and temperate areas of the USA, but the disease is rare in domestic cats. The clinical presentation of feline pythiosis includes subcutaneous masses without ulcerated tissue and involvement of the intestinal tract. Here in we report an eight-week-old female unvaccinated stray kitten with an unusual extensive circular ulcerated lesion on her left flank. The lesion did not respond favorably to administration of systemic antibiotics. Clinical specimens submitted for culture demonstrated submerged fungal-like flat colonies later identified as *Pythium insidiosum*, a finding also confirmed by histopathology, serology, and DNA sequencing and thus, treated with itraconazole. Since no improvement was observed, *Pythium*-immunotherapy was initiated. The cat responded to the latter approach and in less than 10 days, the lesion had contracted around the edges and was crusting off to reveal healthy granulation tissue. Twenty-three days after immunotherapy was initiated the original wound had shrunken significantly to a small scabbed area. However, the cat acutely developed tachypnea, lung and intestinal complications and due to her rapid deterioration, humane euthanasia was elected. Unfortunately, necropsy was not conducted. The clinical presentation reported here suggests large ulcerative cutaneous lesions caused by *P. insidiosum* can also occur in domestic cats. Despite reports of unsuccessful treatment results of feline pythiosis using *Pythium*-immunotherapy, this report suggests this approach might be helpful in similar feline cases.

## 1 Introduction

Feline pythiosis is caused by *Pythium insidiosum* an and oomycete fungal-like pathogen within the SAR (for “stramenopiles, alveolates and rhizaria”) supergroup [1]. Feline pythiosis is rarely reported in the USA or around the world [1,2]. Bissonnette et al. [3] in 1991 first reported feline pythiosis in a 3-year-old domestic cat with nasopharyngeal involvement extending into the orbital cavities. This cat was cured by co-administration of itraconazole and ketoconazole. One year later, Duncan et al. [4] reported four cases of cutaneous feline pythiosis. One of the cats was unsuccessfully treated with itraconazole and *Pythium*-immunotherapy [1], but was later cured only following surgical excision. Clinical data was not available for the other cats.

In 1998 Thomas and Lewis [2] reported two additional cases in cats with large subcutaneous masses without characteristic ulcerative disease reported in other species (dogs and horses) [1]. Following limb amputation, one patient later cured, whereas other cat was initially treated with fluconazole, terbinafine and *Pythium*-immunotherapy without success. Subsequently the cat was treated with a combination of amphotericin B and itraconazole also without response. The authors reported the subcutaneous lesions in the latter case progressed very slowly. Based on these observations they concluded that the invasion of the subcutaneous tissues by *P. insidiosum* “may be slower in cats than in dogs” [2]. More recently, several cases of subcutaneous feline pythiosis were diagnosed in Brazil [5,6] and the USA^7.8^ suggesting clinicians are better recognizing the disease in this species. Herein, we report an unusual case of cutaneous feline pythiosis with an extensive ulcerative lesion on her left flank.

## 2 Case

**Fig. 1:**
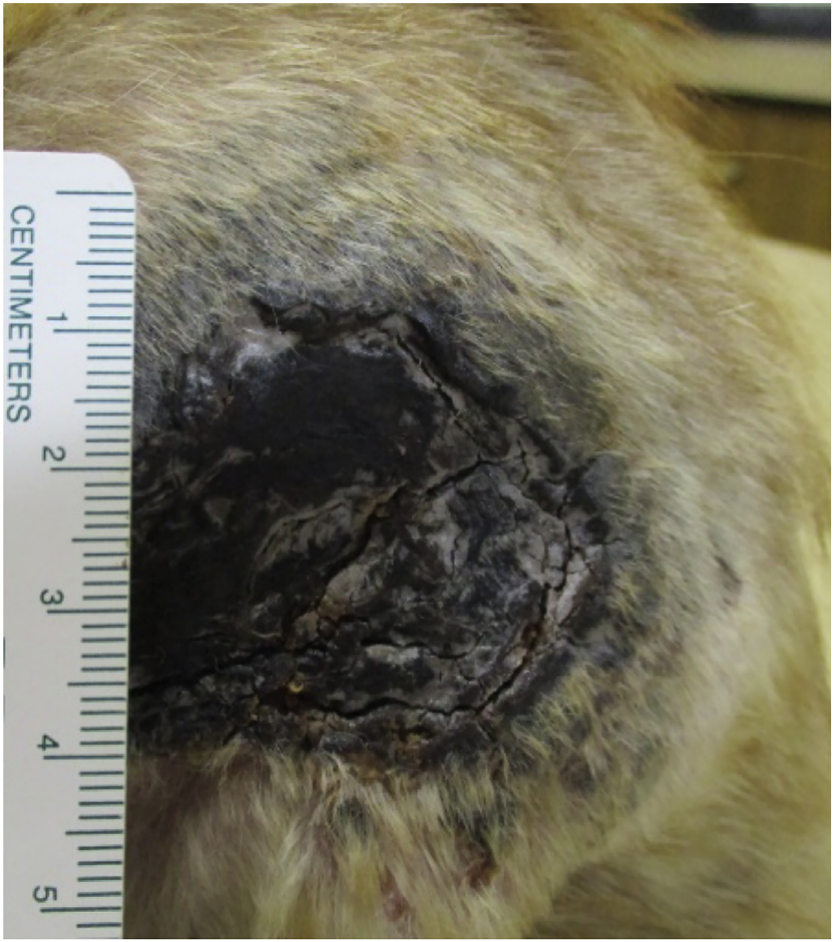
The figure depicts the original round lesions covered by a black necrotic crusted surface first observed at admission.

**Fig. 2:**
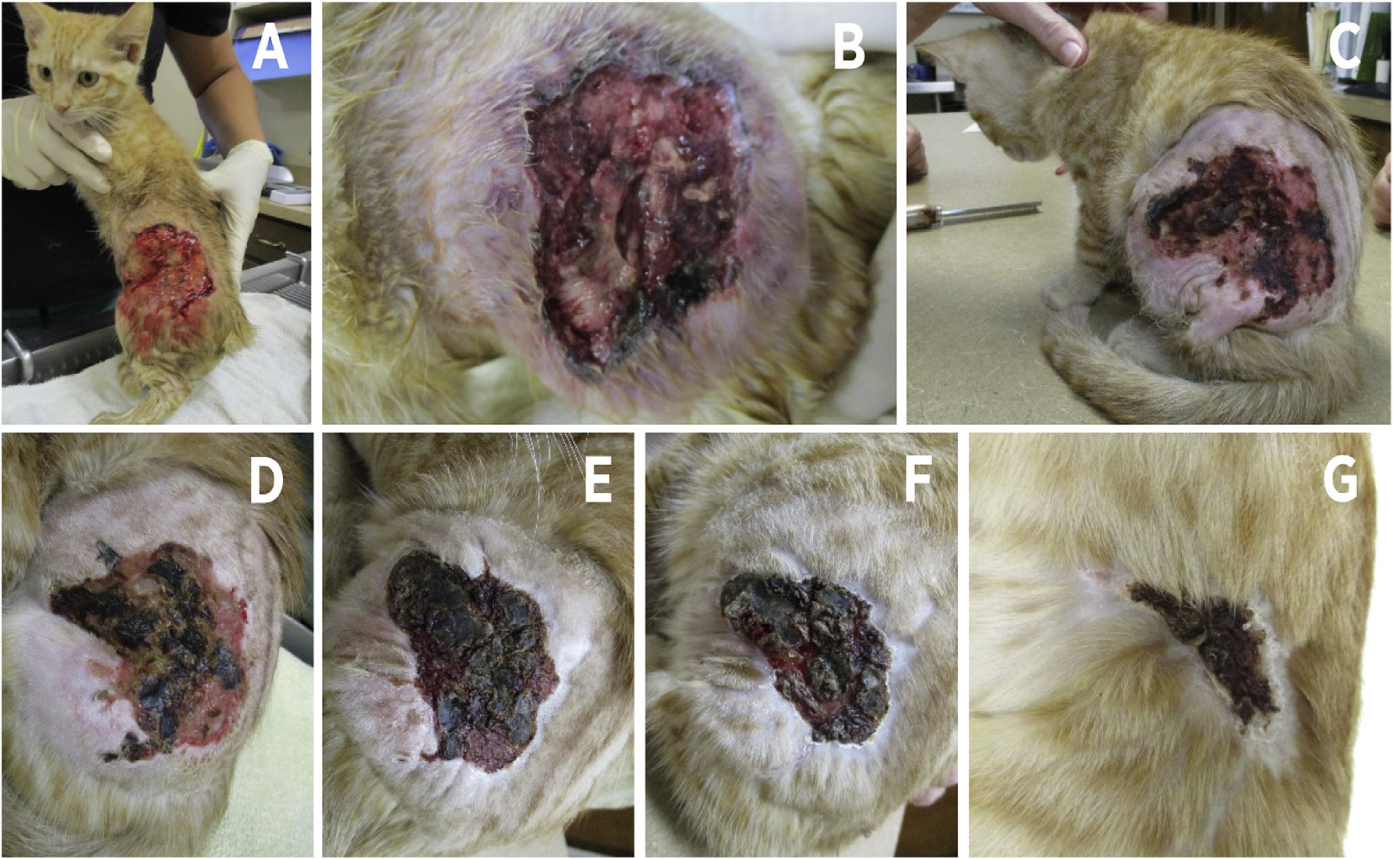
Panel A and B depicts the lesion after removing the black cluster shown in Fig. 1 (before immunotherapy). Panels C to G showed the cat in the following weeks after the first *Pythium*-immunotherapy. Note the formation of healthy tissue around the old lesion. Panel G shows an almost healed lesion. At this point, the cat was considered cured.

An 8-week-old female kitten presented to South Orlando Animal Hospital with a 2-week history of a rapidly growing, round and pruritic lesion on her left flank. She was adopted after being found as a stray cat in the Central Florida area. At presentation, the lesion was circular and approximately 6.0 X 7.0 cm in diameter covered by a necrotic black crusted surface (Fig. 1). Surrounding dermis and subcutaneous tissues were firm and painful upon palpation. The patient was infested with fleas and pyrexic (39.6 °C). The lesion was debrided under sedation, thoroughly cleaned and permitted to heal by second intention (Fig. 2A and B). The kitten was given buprenorphine and Convenia® (cefovecin 3.6 mg/lb) injections subcutaneously and sent home with amoxicillin clavulanate oral drops (62.5 mg twice a day). Owners were instructed to perform hydrotherapy on the wound and keep in a clean environment.

**Fig. 3:**
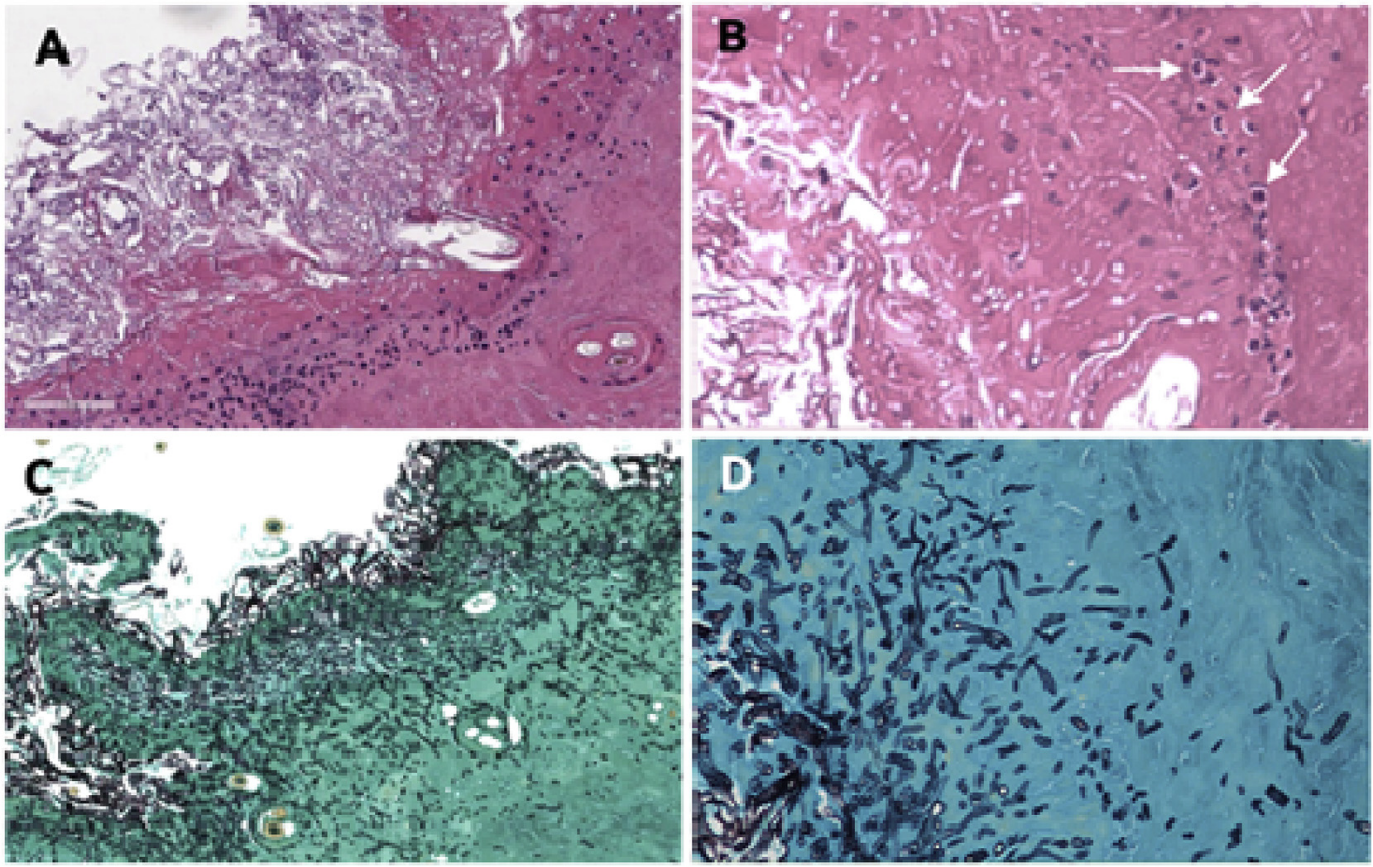
The figure showed the histopathological findings in H&E (Panels A and B). Necrotic tissue and inflammatory infiltrate are observed along with unstained long filaments corresponding to *Pythium insidiosum* hyphae and some eosinophils (arrows). Panels C and D are Gomori Methenamine-Silver (GMS) stains showing the presence of transversally and longitudinal *P. insidiosum* hyphae penetrating deep in the subcutaneous tissues. (Panels A and C= 10X; Panels B and D= 50X).

Two weeks later, the wound showed no improvement so a biopsy was taken from the wound margin for histopathology and culture. On physical exam, the kitten had enlarged axillary lymph nodes bilaterally and remained pyrexic (39.6 °C). The wound was again debrided and a wet-to-dry bandage was applied and changed daily for 3 to 4 days to encourage granulation tissue formation. Amoxicillin clavulanate was continued (as above) and buprenorphine prescribed for pain management (0.4 mg/kg). A serum sample was also collected and sent to Pan American Veterinary Laboratories for an ELISA anti-*P. insidiosum* IgG detection (PanAmVetLabs, Lexinton, Texas. Positive 200%<; Negative 0–150%>; Borderline 151–199%>). Histopathology showed the presence of eosinophilic granulomatous tissue with numerous hyaline slender sparsely septate hyphae (Fig. 3A–D). The organism recovered in culture was identified by DNA sequencing analysis of its ITS1-5.8S-ITS2 (using the universal primer ITS1 and ITS4^13^) as *P. insidiosum* (GenBank accession number= MK772067) and thus, treatment with itraconazole (10 mg/kg q 24 h) was then initiated. Seven days later, the lesion showed no change and the kitten remained pyrexic. Since no improvement was observed with antifungal therapy, all previous treatments were discontinued and *Pythium*-immunotherapy, using 200 μg/ml of purified *P. insidiosum* immunogens, was initiated administering 1.0 mL of these antigens subcutaneously [1]. This decision was further supported by ELISA demonstrating the presence of anti-*P. insidiosum* IgG antibodies (250%).

One week after the first injection, the lesion appeared drier and the crusted surface peeled off, exposing healthy looking granulation tissue and the wound was contracting around its edges (Fig. 2C). The kitten was otherwise clinically normal, no longer pyrexic (38.2 °C) and the axillary lymph nodes returned to normal size. The second injection of *Pythium*-immunotherapy was administered at this time. The kitten improvement was monitored for the following ten days (Fig. 2D–F). Twelve days after the last visit, the kitten presented for labored breathing and inappetence of 2 days duration. Physical exam revealed tachypnea with marked inspiratory effort and dull lung sounds ventrally. The abdomen was moderately distended. The wound was almost fully healed (Fig. 2 G). Radiographs revealed severe pleural effusion and a mass effect within the retroperitoneal space causing ventral displacement of abdominal organs. White/opaque fluid was obtained from the chest via thoracocentesis. Cytological examination of the fluid revealed lymphocytic infiltrate. Culture of the fluid was negative for both bacteria and fungi. Due to financial constraints and grave prognosis, humane euthanasia was elected. Unfortunately, necropsy was not performed.

## 3 Discussion

Cutaneous ulcerative and exudative lesions is a common clinical feature of dogs and horses with cutaneous pythiosis [1,2]. Feline pythiosis is rare and has been diagnosed mostly affecting the subcutaneous or intestinal tissues [2]. Cutaneous ulcerative feline pythiosis had been reported only once by Duncan et al. [4] These authors diagnosed four cases of feline pythiosis, two displaying ulcerative lesions on their limbs and the remaining demonstrating subcutaneous lesions (without ulceration) on their inguinal and umbilical areas respectively. However, few details are available on the clinical presentation of the Duncan et al. [4] cases. Coincidentally, a figure on one of Duncan et al. [4] cats depicting a cutaneous ulcerative lesion was included in the Thomas and Lewis [2] review. This figure showed a protractive single dry ulcerated lesion over the hock of the infected cat. This feature is in contrast with the rapidly progressive proliferative wet lesion observed in the present case. Although pythiosis clinical presentation in other species [1], including humans [9] follow a progressive clinical feature with cutaneous ulcers and draining tracts, most cases so far reported in cats tend to be clinically protractive and restricted to the subcutaneous and/or intestinal tissues [1,7]. However, the clinical findings observed in this case suggests feline pythiosis may also include fast-growing cutaneous ulcerative lesions that seem to respond better to *Pythium*-immunotherapy but not to itraconazole.

At the time of adoption, the cat had already developed a circular ulcerative lesion over her left flank. Since she was a stray cat, vaccination against several feline diseases was to be part of the initial protocol. However, priority was to first treat the cutaneous lesion and thus, vaccination was delayed. In addition, it was not known whether there would be interference between injections with *Pythium*-immunotherapy. Ten days after the first immunotherapeutic injection the cat was in good health and the early symptoms such as pain at the site of the lesion, fever and enlarged lymph nodes had subsided. Thus, when returned with acute respiratory and intestinal problems, the possibility that the cat had contracted a viral infection that rapidly involved the respiratory and the intestinal tracts was suggested. However, we do not rule out the possibility that somehow, the manipulation of the immune response from a strong Th2 to a Th1, a feature of *Pythium*-immunotherapy [1], may have had an adverse reaction, contributing the cats’ demise.

Feline pythiosis had also been diagnosed in captive big cats affecting intestinal and pulmonary tissues [9,10]. Furthermore, species of *Lagenidium* such as *L. deciduum* (*L. vilelae*), another oomycete phylogenetically and taxonomically related to *P. insidiosum*, was also diagnosed causing protractive subcutaneous infections in cats with clinical and histopathological features similar to that in feline pythiosis [[11], [12], [13]]. Although *Lagenidium* hyphae is broader than that displayed by *P. insidiosum*, the clinical and histopathological features of both infections are comparable [11]. In addition, both oomycetes cause protractive subcutaneous lesions and the formation of eosinophilic granulomas with the presence of hyphae at the center of micro abscesses surrounded by eosinophils in cats [11,13].

## 4 Conclusions

Although the clinical features of previously reported feline pythiosis have displayed protractive cutaneous or subcutaneous lesions, the current case suggests rapidly progressive ulcerative cutaneous lesions can also be expected in this species. In addition, the management of feline pythiosis is difficult with only a few reportedly cured cases using surgery and antifungal drugs without *Pythium*-immunotherapy [[2], [3], [4],^8^]. The cat in this report is the first to respond to this approach, and thus this therapeutic tool should be considered as part of the treatment choices in cases suspected of feline pythiosis.

## Declaration of competing interest

There are not.
